# Performance of the modified 2022 ACR/EULAR giant cell arteritis classification criteria without age restriction for discriminating from Takayasu arteritis

**DOI:** 10.1186/s13075-025-03486-y

**Published:** 2025-01-31

**Authors:** Takahiko Sugihara, Masayoshi Harigai, Haruhito A. Uchida, Hajime Yoshifuji, Yasuhiro Maejima, Jun Ishizaki, Yoshiko Watanabe, Hiroaki Dobashi, Yoshinori Komagata, Naoto Tamura, Yoshikazu Nakaoka, Japan Research Committee of the Ministry of Health, Labour, and Welfare for Intractable Vasculitis (JPVAS), Yoshiya Tanaka, Tsutomu Takeuchi, Taio Naniwa, Hiroko Nagafuchi, Takahiro Okazaki, Tetsuya Horita, Tatsuya Atsumi, Yoshihiro Arimura, Mitsuaki Isobe, Kazuo Tanemoto, Noriyoshi Ogawa, Yohko Murakawa, Shunsuke Furuta, Hitoshi Hasegawa, Yasuhiro Katsumata, Eisuke Amiya, Hiroshi Akazawa, Issei Komuro, Koichi Amano, Atsushi Kawakami, Shigeto Kobayashi, Takashi Wada, Eri Muso, Atsushi Komatsuda, Satoshi Ito, Noriyuki Homma, Taichi Hayashi, Shinichi Takeda, Takashi Wada

**Affiliations:** 1 Department of Rheumatology, Graduate School of Medical and Dental Sciences, Institute of Science Tokyo, Tokyo, Japan; 2 Division of Rheumatology, Department of Internal Medicine, Toho University School of Medicine, 6-11-1 Omori-Nishi, Ota-Ku, Tokyo, 143-8541 Japan; 3 Division of Rheumatology, Department of Internal Medicine, Tokyo Women’s Medical University School of Medicine, Tokyo, Japan; 4 Department of Chronic Kidney Disease and Cardiovascular Disease, Dentistry and Pharmaceutical Sciences, Okayama University Graduate School of Medicine, Okayama, Japan; 5 Department of Rheumatology and Clinical Immunology, Graduate School of Medicine, Kyoto University, Kyoto, Japan; 6 Department of Cardiovascular Medicine, Graduate School of Medical and Dental Sciences, Institute of Science Tokyo, Tokyo, Japan; 7 Department of Hematology, Clinical Immunology and Infectious Diseases, Ehime University Graduate School of Medicine, Ehime, Japan; 8 Department of General Medicine, Kawasaki Medical School, Kurashiki, Japan; 9 Division of Hematology, Rheumatology and Respiratory Medicine, Department of Internal Medicine, Faculty of Medicine, Kagawa University, Kagawa, Japan; 10 Department of Nephrology and Rheumatology, Kyorin University School of Medicine, Tokyo, Japan; 11 Department of Internal Medicine and Rheumatology, Juntendo University School of Medicine, Tokyo, Japan; 12 Department of Vascular Physiology, National Cerebral and Cardiovascular Center Research Institute, Suita, Japan; 13 Department of Cardiovascular Medicine, National Cerebral and Cardiovascular Center Hospital, Suita, Japan; 14 Department of Cardiovascular Medicine, Osaka University Graduate School of Medicine, Suita, Japan

**Keywords:** GCA, TAK, Classification criteria, Bilateral axillary artery, Descending thoracic-abdominal aorta, Bilateral subclavian artery

## Abstract

**Objective:**

To evaluate the ability to discriminate giant cell arteritis (GCA) from Takayasu arteritis (TAK) according to the modified 2022 American College of Rheumatology/European Alliance of Associations for Rheumatology (ACR/EULAR) GCA classification criteria.

**Methods:**

Patients enrolled in the Japanese nationwide retrospective registry were evaluated using the criteria with partial modification; wall thickening of descending thoracic-abdominal aorta were mainly diagnosed by contrast-enhanced computed tomography (CT) or magnetic resonance imaging instead of evaluating with positron emission tomography (PET)-CT. The discriminability of the criteria was evaluated using C-statistic (> 0.7: good ability).

**Results:**

Newly diagnosed patients with GCA (*n* = 139) and TAK (*n* = 129) were assessed, and 23.3% of TAK were aged 50 years or older at onset. The sensitivity of the modified 2022 ACR/EULAR GCA classification criteria with a score ≥ 6 was 82.0%, 68.5%, and 32.1% in all GCA, GCA with large-vessel involvement, and GCA without cranial arteritis, respectively. The specificity of the modified criteria was 96.1% for the 129 TAK as controls. Five patients with late-onset TAK met the modified criteria, and four had cranial signs and symptoms, two had bilateral axillary artery involvement, and four had descending thoracic-abdominal aorta involvement. The discriminability of the criteria was good (C-statistic: 0.986, 95% confidence interval [CI]: 0.976–0.996) and remained good after excluding age (C-statistic: 0.927, 95% CI: 0.894–0.961). The discriminability of a set of large-vessel lesions (bilateral axillary artery and descending thoracic-abdominal aorta) and inflammatory markers was markedly decreased with poor C-statistic value (C-statistic: 0.598, 95% CI: 0.530–0.667). Discriminability was improved after adding polymyalgia rheumatica (PMR) (C-statistic: 0.757, 95% CI: 0.700–0.813) or age (C-statistic: 0.913, 95%CI: 0.874–0.951) to the set of large-vessel lesions. In GCA patients with a score ≤ 5, 52% had bilateral subclavian and/or axillary artery involvement.

**Conclusion:**

The modified 2022 ACR/EULAR GCA classification criteria well performed in classifying GCA and TAK without PET-CT in routine clinical practice. A set of items included in the modified GCA classification criteria had good discriminative ability for GCA and TAK, even when age was excluded. However, age restriction or PMR was required to distinguish GCA without cranial lesions from TAK.

**Supplementary Information:**

The online version contains supplementary material available at 10.1186/s13075-025-03486-y.

## Backgrounds

Temporal artery biopsy is a test with high specificity for the diagnosis of GCA [1, 2]. However, diagnostic yield depends on timing after treatment initiation and correct sampling, and the diagnostic sensitivity of biopsy is 30–50% [3]. The 1990 ACR criteria for the classification of GCA consist of clinical features of temporal arteritis and a positive temporal artery biopsy [4]. Recent advances in imaging technology revealed that patients with GCA frequently have lesions in the aorta and its major branches, and some of these patients do not have temporal artery involvement [5].

Previous studies have evaluated the similarities and differences between Takayasu arteritis (TAK) and GCA [6–8]. A cluster analysis of the patients from the Diagnostic and Classification Criteria for Vasculitis (DCVAS) cohort and a combined North American cohort showed different patterns of vascular involvement between TAK and GCA [9]. This finding led to the development of the 2022 ACR/EULAR classification criteria for GCA [10].

The Japan Research Committee of the Ministry of Health, Labour, and Welfare for Intractable Vasculitis (JPVAS) performed a nationwide, multicenter, retrospective cohort study of patients with GCA and TAK [11–13]. The clinical features of Japanese patients newly diagnosed with GCA were similar to those of Western countries, and large-vessel lesions were detected in approximately half of the patients [11].

Since GCA and TAK share common clinical signs and symptoms related to large-vessel lesions, it is important to validate the ability to discriminate GCA from TAK according to the 2022 ACR/EULAR GCA classification criteria, especially in a country like Japan where TAK is more prevalent compared to Europe and the United States. In this study, we evaluated the sensitivity and specificity of the modified 2022 ACR/EULAR GCA classification criteria in the JPVAS GCA/TAK cohort. Since late-onset TAK is prevalent in Japan [14, 15], we evaluated the discriminative ability of the items, except age, in the modified 2022 ACR/EULAR GCA classification criteria. We also evaluated the clinical characteristics of GCA not meeting the modified 2022 ACR/EULAR GCA classification criteria.

## Methods

### JPVAS study group and GCA/TKA cohort

The JPVAS is a study group on vasculitis supported by the Ministry of Health, Labour and Welfare of Japan. The study group implemented a nationwide, multicenter, retrospective cohort study of newly-diagnosed patients with GCA or TAK at 23 participating institutions between 2007 and 2014 [11, 13]. All incident cases were enrolled in the JPVAS cohort. Patients were not involved in the design and conduct of this research. The Ministry of Health, Labour and Welfare of Japan diagnostic criteria for TAK [16] were adopted for patients with TAK in the JPVAS cohort, which permitted the enrollment of patients with early-stage TAK having multiple or diffuse lesions of wall thickening, even in the absence of stenosis and dilatation of large-vessel lesions as well as late-onset TAK with onset at age 50 years or older, which differ from the 2022 ACR/EULAR TAK classification criteria [17]. For the diagnosis of GCA and TAK, medical records were reviewed retrospectively by experts in vasculitis practice at JPVAS, and eligible patients were classified as GCA or TAK according to the judgment of experts at each institution specialized in vasculitis, and no classification criteria were used to differentiate between GCA and TAK in the present cohort. When the steering committee of this study reevaluated the diagnosis of the enrolled patients, there was a risk that the decision would be influenced by the 2022 ACR/EULAR classification criteria for GCA and TAK. Therefore, the diagnosis of experts in vasculitis affiliated with JPVAS was used as the gold standard for the diagnosis in this study. We collected patient information using the same case report form on systemic symptoms, clinical features associated with cranial arteritis, clinical features associated with large-vessel lesions, PMR, inflammatory markers, and large-vessel imaging. We also assessed the histopathological findings of the temporal artery in patients with GCA.

### Assessments

Patients were assessed for the following findings. Cranial features included a new headache, abnormal examination of the temporal artery, scalp tenderness, sudden visual loss or abnormalities, jaw claudication, and a positive temporal artery biopsy.Musculoskeletal features included PMR and myalgia/arthralgia/arthritis. Inflammatory markers included ESR and CRP. Aortic regions evaluated by imaging study included the subclavian artery, axillary artery, carotid artery, vertebral artery, brachiocephalic artery, ascending aorta, aortic arch, descending thoracic aorta, abdominal aorta, renal artery, mesenteric artery, iliac artery, and femoral artery. These lesions were detected by enhanced computed tomography (CT)/CT angiography (CTA), enhanced magnetic resonance imaging (MRI)/magnetic resonance angiography (MRA), or ultrasonography. In the JPVAS cohort, CT/CTA, MRI/MRA, or ultrasonography were mainly used to assess large-vessel lesions of GCA and TAK. Imaging examinations of large-vessel lesions were performed in 135 of the 139 patients at diagnosis of GCA [11] and in all cases of TAK [13]. Japanese insurance covers positron emission tomography (PET) only for evaluating disease activity in confirmed cases with GCA or TAK, and 38 or 50 received PET-CT at the diagnosis of GCA [11] or TAK [13], respectively.

### Scoring using the modified 2022 ACR/EULAR classification criteria for GCA

Table 1 shows the different assessments performed using the 2022 ACR/EULAR GCA classification criteria [10] and the modified 2022 ACR/EULAR GCA classification criteria in the present study. Patients with a halo sign on temporal artery ultrasound had a score of 5 points in the 2022 ACR/EULAR classification criteria for GCA, but we did not assess the presence of a halo sign because the present retrospective cohort was not constructed for the purpose of evaluating the 2022ACR/EULAR classification criteria and because temporal artery lesions were primarily evaluated by temporal artery biopsy in GCA patients diagnosed between 2007 and 2014.

**Table 1:** Assessments used in the 2022 ACR/EULAR GCA classification criteria and the modified version in this study

2022 ACR/EULAR GCA classification criteria	Modified ACR/EULAR GCA classification criteria in the present study	Score
Absolute requirement		
Age 50 years at time of diagnosis	Unmodified	
Additional clinical criteria		
Morning stiffness in the shoulders/neck	PMR	+ 2
Sudden visual loss	Sudden visual abnormalities or visual loss	+ 3
Jaw or tongue claudication	Jaw claudication	+ 2
New temporal headaches	New headaches not explained by other diseases	+ 2
Scalp tenderness	Unmodified	+ 2
Abnormal examination of the temporal artery	Unmodified	+ 2
Laboratory, imaging, and biopsy criteria		
Maximum ESR ≥ 50 mm/hour or maximum CRP ≥ 10 mg/L	Unmodified	+ 3
Positive temporal artery biopsy or halo sign on temporal artery Ultrasound	Positive temporal artery biopsy	+ 5
Bilateral axillary involvement	Unmodified	+ 2
FDG‐PET activity throughout Aorta	Wall thickening or aneurysm on CT/CTA or MRI/MRA, and/or PET-CT activity throughout the aorta	+ 2

*ACR* American College of Rheumatology, *CTA* computed tomography angiography, *FDG* fluorodeoxyglucose, *JPVAS* Japan Research Committee of the Ministry of Health, Labour, and Welfare for Intractable Vasculitis, *MRA* magnetic resonance angiography

Japanese health insurance covers positron emission tomography (PET) only for assessing disease activity in confirmed cases with GCA or TAK by CT, MRI, or ultrasonography. Therefore, we needed to modify the classification criteria. In the modified 2022 ACR/EULAR GCA classification criteria, we assessed the presence of wall thickening in the descending thoracic aorta-abdominal aorta (The involvement of both the descending thoracic aorta and the abdominal aorta) on enhanced CT, enhanced MRI, ultrasonography, or fluorodeoxyglucose (FDG)-PET-CT instead of FDG-PET activity throughout the aorta using the 2022 ACR/EULAR classification criteria for GCA; patients with this feature had a score of 2 points.

A score ≥ 6 points in total using the above scoring system denoted GCA based on the 2022 ACR/EULAR classification criteria.

### Statistical analysis

For comparison of GCA and TAK or late-onset TAK, continuous variables were evaluated by using Student’s *t*-test or the Mann–Whitney *U* test and categorical variables were examined by using the chi-squared test and Fisher’s exact test. For comparison between the three groups, continuous variables were analyzed through a one-way analysis of variance and Tukey’s multiple tests. Categorical variables were evaluated by multiple tests using the Z-test.

To calculate C statistics, we used multivariable logistic regression analysis to evaluate associations between GCA and items of the classification criteria. The predicted probability that a set of classification criteria items of interest could accurately diagnose GCA was recorded for each patient by using the logistic model. Moreover, the area under the receiver operating characteristic curve was calculated, as a C-statistic value [18]. A value for the C-statistic > 0.7 indicates a reasonable or good discriminative ability. All analytical procedures were performed using IBM SPSS Version 26.0 (IBM Corp., Armonk, NY, USA). All reported *p*-values were two-tailed, and the level of significance was set to < 0.05.

## Results

### Comparison of clinical parameters included in the 2022 ACR/EULAR GCA classification criteria in patients with GCA and TAK

Newly diagnosed patients with GCA (*n* = 139) and TAK (*n* = 129) were assessed (Table 2). The proportions of patients with cranial signs and symptoms (new headaches, scalp tenderness, abnormal examination of the temporal artery, sudden visual abnormalities, and jaw claudication), PMR, and myalgia/arthralgia/arthritis were significantly higher in GCA versus TAK (Table 2). Of the 139 patients with clinically diagnosed GCA, temporal artery biopsy was conducted in 87 (62.6%), and 70 (50.4%) had positive pathological findings in the temporal artery biopsy. Of the 139 patients with GCA, any large-vessel involvement by imaging was detected in 52.5%. Bilateral axillary arteries were observed in 10 patients (7.2%); eight patients were diagnosed with PET-CT; one patient had wall thickening on CTA; and one patient had arterial stenosis on CTA. Descending thoracic to abdominal aortic lesions were observed in 33 patients (23.7%); 32 patients were diagnosed with wall thickening by CTA or MRA (CTA: *n* = 27; MRA: *n* = 15) and 22 of them also had FDG uptake confirmed by PET-CT; and one patient was diagnosed by PET-CT alone. The proportions of bilateral axillary artery involvement and lesions of descending thoracic aorta-abdominal aorta were similar in GCA and TAK (Table 2). The proportion of bilateral axillary artery involvement (13.7%) and lesions of descending thoracic aorta-abdominal aorta (45.2%) in large-vessel GCA (LV-GCA) was numerically higher than those in TAK (Supplementary Table S1). The increase in odds ratio (OR) for LV-GCA of bilateral axillary artery involvement (OR:2.40; 95% confidence interval [95%CI]: 0.90–6.39) and descending thoracic aorta-abdominal aorta (OR:1.71, 95%CI: 0.95–3.08) was not statistically significant but showed a higher trend compared to ORs of unilateral left or right subclavian artery lesions, ascending aorta, aortic arch, aortic involvement of ≥ two lesions (Supplementary Table S1).

**Table 2:** Comparison of clinical parameters included in the modified 2022 ACR/EULAR GCA classification criteria in patients with GCA and TAK

	GCA (*n* = 139)	TAK (*n* = 129)	*P*-value
Age at onset	73.8 (7.7)	35.4 (18.1)	< 0.001
Age at onset ≥ 50 years, %	100	23.3	< 0.001
Female, %	66.9	83.7	0.001
New headache, %	61.2	15.9 (18/113)	< 0.001
Scalp tenderness, %	19.4	0 (0/105)	< 0.001
Abnormal examination of the temporal artery, %	59.0	0.9 (1/110)	< 0.001
Sudden visual loss, %	23.7	7.1 (8/113)	< 0.001
Jaw claudication, %	36.0	5.5 (6/110)	< 0.001
PMR, %	41.7	0.8 (1/127)	< 0.001
Myalgia/arthralgia/arthritis, %	56.8	20.5 (25/122)	< 0.001
Definitive vasculitis on temporal artery biopsy	50.4	0	-
Stenosis of temporal artery by imaging	26.6	0	-
ESR ≥ 50 mm/hr or CRP ≥ 1.0 mg/dL, %	95.7	83.7	0.001
Large-vessel involvement, %	52.5	100	-
Left axially artery, %	10.8	12.4	0.680
Right axially artery, %	8.6	7.0	0.614
Bilateral axillary artery, %	7.2	6.2	0.746
Left subclavian artery, %	29.5	64.3	< 0.001
Right subclavian artery, %	23.7	36.4	0.023
Bilateral subclavian and/or axillary artery^a^, %	21.6	30.2	0.106
Left or right subclavian and/or axillary artery, %	31.7	70.5	< 0.001
Descending thoracic aorta-abdominal aorta^b^, %	23.7	32.6	0.108
Ascending aorta	16.5	48.8	< 0.001
Aortic arch	25.2	57.4	< 0.001
Aortic involvement of ≥ two lesions	27.3	58.9	< 0.001

Categorical variables were examined by using the chi-squared test. Continuous variables were evaluated by using the Student’s *t*-test. Odds ratios of being diagnosed with GCA compared to TAK were calculated

*TAK* Takayasu arteritis

^a^The presence of lesions of the subclavian and/or axillary arteries on both the left and right sides. The case in which one artery was the subclavian artery and the other was the axillary artery was also included

^b^The involvement of both the descending thoracic aorta and the abdominal aorta

### Comparison of clinical parameters included in the modified 2022 ACR/EULAR GCA classification criteria in patients with LV-GCA and late-onset TAK

In the JPVAS cohort, 23.3% of TAK cases had a late onset at age 50 years and older, and a similar trend was observed when comparing LV-GCA to late-onset TAK. Cranial signs and symptoms, PMR, and bilateral subclavian and/or axillary artery involvement skewed towards LV-GCA rather than late-onset TAK. Whereas, ascending aorta were towards late-onset TAK. However, the incidences of axillary arteries and descending thoracic-abdominal aorta in late-onset TAK were similar to those of LV-GCA (Table 3).

**Table 3:** Comparison of clinical parameters included in the modified 2022 ACR/EULAR GCA classification criteria in patients with LV-GCA and late-onset (≥ 50 years) TAK

	LV-GCA (*n* = 73)	Late-onset TAK (*n* = 30)	*P*-value
Age, mean (S.D.)	71.1 (7.1)	64.2 (9.0)	0.001
Female, %	72.6	86.7	0.125
New headache, %	39.7	11.5 (3/26)	0.008
Scalp tenderness, %	15.1	0 (0/24)	0.052
Abnormal examination of the temporal artery, %	41.1	0.0 (0/24)	< 0.001
Sudden visual loss, %	12.3	8.0 (2/25)	< 0.001
Jaw claudication, %	23.3	12.0 (3/25)	0.227
PMR, %	27.4	0.0 (0/28)	< 0.001
Myalgia/arthralgia/arthritis, %	49.3	32.1 (9/28)	0.120
CRP, mg/dL, mean (S.D.)	7.1 (5.1)	8.1 (7.2)	0.513
Left axially artery, %	20.5	13.3	0.391
Right axially artery, %	16.4	13.3	0.474
Bilateral axillary artery, %	13.7	10.0	0.440
Left subclavian artery, %	56.2	70.0	0.192
Right subclavian artery, %	45.2	53.3	0.453
Bilateral subclavian and/or axillary artery^a^, %	41.1	20.0	0.041
Left or right subclavian and/or axillary artery, %	60.3	76.7	0.113
Descending thoracic aorta-abdominal aorta^b^, %	45.2	40.0	0.628
Ascending aorta	31.5	53.3	0.038
Aortic arch	47.9	63.3	0.155
Aortic involvement of ≥ two lesions	52.1	66.7	0.174

Imaging examinations of large-vessel lesions were performed in 135 of the 139 patients at diagnosis of GCA, and 73 had large-vessel lesions as diagnosis of LV-GCA. Categorical variables were examined by using the chi-squared test and Fisher’s exact test. Continuous variables were evaluated by using the Student’s *t*-test

*ACR* American College of Rheumatology, *CRP* C-reactive protein, *ESR* erythrocyte sedimentation rate, *EULAR* European Alliance of Associations for Rheumatology, *GCA* giant cell arteritis, *PMR* polymyalgia rheumatic, *TAK* Takayasu arteritis

^a^The presence of lesions of the subclavian and/or axillary arteries on both the left and right sides. The case in which one artery was the subclavian artery and the other was the axillary artery was also included

^b^The involvement of both the descending thoracic aorta and the abdominal aorta

### Sensitivity and specificity of the modified 2022 ACR/EULAR classification criteria for GCA

Among the 139 patients clinically diagnosed as having GCA (GCA cohort), 114 (82.0%) satisfied the modified 2022 ACR/EULAR GCA classification criteria, and 109 (78.4%) met the 1990 ACR GCA classification criteria (Table 4). In the 129 patients clinically diagnosed as having TAK (TAK cohort), five late-onset patients met the modified 2022 ACR/EULAR GCA classification criteria. Four of the five late-onset TAK had cranial signs and symptoms (headache in one, visual disturbance in two, jaw claudication in two), two had bilateral axillary artery involvement, four had descending thoracic-abdominal aorta involvement, three had ascending aorta and aortic arch involvement, and three had bilateral subclavian artery involvement. Two late-onset TAK with bilateral axillary artery involvement had also ascending aorta and aortic arch involvement. PMR was not reported in the all five late-onset TAK. Overall, the modified 2022 ACR/EULAR GCA classification criteria exhibited a specificity of 96.1%, and the specificity decreased to 83.3% for the 30 patients with late-onset TAK.

**Table 4:** Sensitivity and specificity of the modified 2022 ACR/EULAR classification criteria for GCA in the JPVAS cohort

Classification criteria	Modified 2022 ACR/EULAR^a^	1990 ACR^b^
Sensitivity (GCA, *n* = 139), %	82.0	78.4
Sensitivity (GCA with cranial arteritis, *n* = 111), %	94.6	98.1
Sensitivity (GCA without cranial arteritis, *n* = 28), %	32.1	9.7
Sensitivity (GCA with large-vessel involvement, *n* = 73)	68.5	58.9
Specificity (TAK cohort, *n* = 129), %	96.1	100

*JPVAS* Japan Research Committee of the Ministry of Health, Labour, and Welfare for Intractable Vasculitis, *TAK* Takayasu arteritis

^a^The modified 2022 ACR/EULAR GCA classification criteria

^b^1990 ACR classification criteria for GCA

Among the 111 patients in the GCA cohort with cranial arteritis, 105 (94.6%) met the modified 2022 ACR/EULAR GCA classification criteria, and 106 (98.1%) met the 1990 ACR GCA classification criteria. Among the 73 GCA patients with large-vessel lesions and 28 GCA patients who did not have cranial arteritis, 50 (68.5%) and nine (32.1%) met the modified 2022 ACR/EULAR GCA classification criteria, and 43 (58.9%) and three (9.7%) met the 1990 ACR GCA classification criteria, respectively (Table 4).

### Discriminability of items adapted to the modified 2022 ACR/EULAR GCA classification criteria, excluding age

**Fig. 1:**
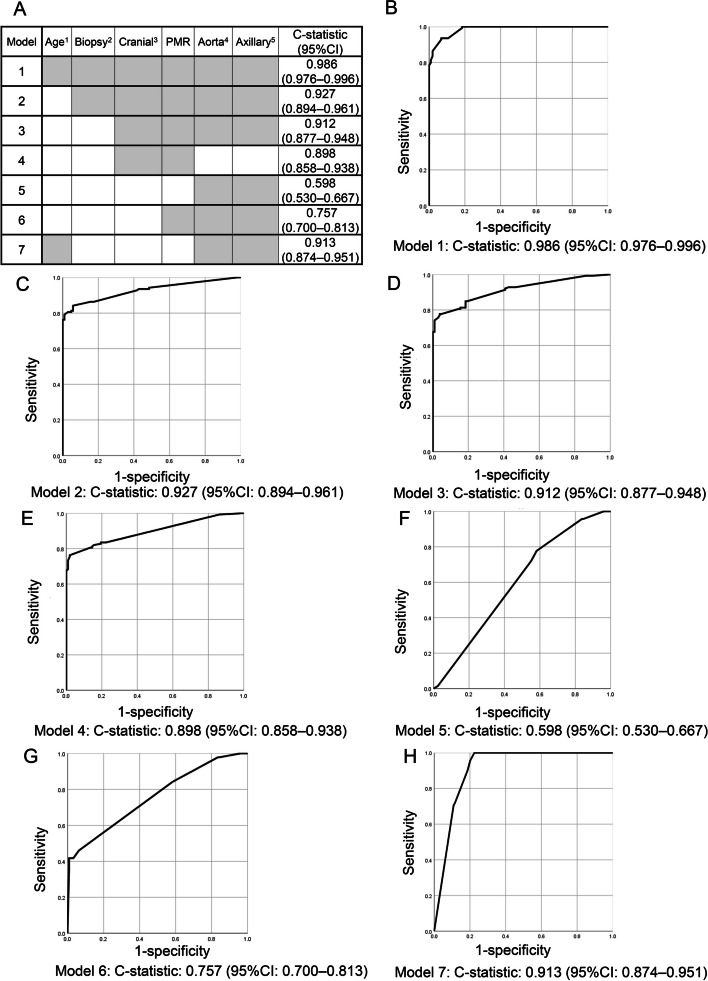
The discriminating ability for GCA and TAK of a combination of clinical parameters was evaluated by C-statistic. Gray highlighting indicates that the item is included in the model 1–7 (**A**). ESR of ≥ 50 mm/hr or CRP of ≥ 1.0 mg/dL was included in all models. Age at onset ≥ 50 years^1^, positive temporal artery biopsy^2^, cranial signs and symptoms (new headache, scalp tenderness, abnormal examination of the temporal artery, sudden visual abnormalities, and jaw claudication)^3^, PMR, descending thoracic-abdominal aorta involvement^4^, bilateral axillary artery involvement^5^, bilateral subclavian and/or axillary artery involvement^6^ were selected as clinical parameters. The ROC curve was drawn to calculate the area under the ROC curve as a C-statistic. The C statistic represents the ability to discriminate GCA from TAK in model 1 (**B**), model 2 (**C**), model 3 (**D**), model 4 (**E**), model 5 (**F**), model 6 (**G**), and model 7 (**H**). A value for the C-statistic greater than 0.7 indicates a reasonable and good ability to discriminate between patients with GCA and TAK. CI, confidence interval; ROC, receiver operating characteristic; TAK, Takayasu arteritis

We calculated the C-statistic to evaluate the discriminative ability of a combination of clinical parameters. Firstly, we evaluated the discriminative ability of a set of all items adopted in the modified 2022 ACR/EULAR GCA classification criteria as Model 1. This set included age ≥ 50 years, a new headache, scalp tenderness, abnormal examination of the temporal artery, sudden visual abnormalities, jaw claudication, PMR, a positive temporal artery biopsy, bilateral axillary artery involvement, descending thoracic-abdominal aorta involvement, and ESR ≥ 50 mm/h or CRP ≥ 1 mg/dL; the C-statistic value was 0.986 (95% CI: 0.976–0.996) (Fig. 1A and B). Even after the exclusion of age from Model 1 (Model 2), the discriminative ability of the combination of clinical parameters (excluding age) was good (C-statistic: 0.927, 95% CI: 0.894–0.961) (Fig. 1A and C). The C-statistic value was still keeping good discriminative ability after excluding a positive temporal artery biopsy from Model 2 (i.e., C-statistic > 0.7) (Fig. 1A and D). Following the exclusion of imaging findings related to large-vessel lesions from Model 3 (Model 4), the discriminative ability of clinical criteria regarding cranial signs and symptoms remained good (Fig. 1A and E).

### Discriminative ability of items of large-vessel lesions in the modified 2022 ACR/EULAR GCA classification criteria, excluding age

In the presence of cranial signs and symptoms, the differential diagnosis between GCA and TAK is straightforward. Therefore, we evaluated the discriminative ability of a set of items excluding both cranial signs and symptoms and PMR. Initially, we assessed the ability of a set of imaging findings (i.e., bilateral axillary artery involvement and descending thoracic-abdominal aorta involvement), and ESR ≥ 50 mm/h or CRP ≥ 1 mg/dL (Model 5); the C-statistic value was markedly reduced to 0.598 (95%CI: 0.530–0.667) (Fig. 1A and F). Following the addition of PMR to Model 5 (Model 6), the discriminating power was improved (C-statistic: 0.757: 95%CI: 0.700–0.813) (Fig. 1A and G), and it was good discriminative ability (i.e., C-statistic > 0.7). The addition of age to Model 5 (Model 7) also improved the discriminative ability (C-statistic: 0.913, 95%CI: 0.874–0.951) (Fig. 1A and H).

### Clinical features of patients who failed to meet the modified 2022 ACR/EULAR GCA classification criteria

Table 5 shows the clinical features of 25 patients who failed to meet the modified 2022 ACR/EULAR GCA classification criteria (i.e., score ≤ 5). The proportions of cranial signs and symptoms and PMR were significantly lower in GCA patients with a score ≤ 5 than in GCA patients with a score ≥ 6; these proportions were similar to those recorded for TAK. Imaging findings revealed that 92.0% of the 25 patients with GCA with a score ≤ 5 had any large-vessel involvement. However, the proportion of bilateral axillary artery involvement was 0%, while the proportion of bilateral subclavian and/or axillary artery involvement was 52% in GCA patients with a score ≤ 5, which was the highest among the three groups (Table 5). The proportion of descending thoracic-abdominal aorta involvement in GCA patients with a score ≤ 5 was similar to that recorded in GCA patients with a score ≥ 6 and TAK.

**Table 5:** Clinical features of patients who failed to meet the modified 2022 ACR/EULAR classification criteria of GCA

	GCA (score ≥ 6) (*n* = 114)	GCA (score ≤ 5) (*n* = 25)	TAK (*n* = 129)
Age	74.6 (7.2)	70.2 (8.9)	35.4 (18.1)^**^
Age at onset ≥ 50 years, %	100	100	23.3^*^
Female, %	64.0	80.0	83.7^**^
New headache, %	71.1^*^	16.0	15.9
Scalp tenderness, %	23.7^*^	0	0
Abnormal examination of the temporal artery, %	71.1^*^	4.0	0.9
Sudden visual loss, %	28.1^*^	4.0	7.1
Blindness	5.3^*^	0	0
Jaw claudication, %	43.9^*^	0	5.5
PMR, %	48.2^*^	12.0^*^	0.8^*^
Myalgia/Arthralgia/Arthritis, %	60.5^***^	40.0	20.5
CRP	8.12 (5.76)	6.42 (5.74)	6.13 (5.45)
ESR	92.6 (33.3)	73.1 (36.8)	74.0 (36.8)
ESR ≥ 50 mm/hr or CRP ≥ 1.0 mg/dL, %	97.4^***^	87.5	83.7
Large-vessel involvement	43.9^*^	92.0^*^	100^*^
Left axially artery, %	10.5	12.0	12.4
Right axially artery, %	9.6	4.0	7.0
Bilateral axillary artery, %	8.8	0	6.2
Left subclavian artery, %	21.1^*^	68.0	64.3
Right subclavian artery, %	17.5^*^	52.0	36.4
Bilateral subclavian and/or axillary artery^a^, %	14.9^*^	52.0	30.2
Left or right subclavian and/or axillary artery, %	23.7^*^	68.0	70.5
Descending thoracic aorta-abdominal aorta^b^, %	21.7	33.3	32.6
Ascending aorta	12.3^*^	36.0	48.8
Aortic arch	19.3^*^	52.0	57.4

Continuous variables are determined by one-way ANOVA and Turky’s multiple test. Categorical variables are determined by multiple tests using the Z-test

*LV-GCA* large-vessel giant cell arteritis, *OR* odds ratios, *TAK* Takayasu arteritis

^*^p < 0.05 compared to the other two groups

^**^p < 0.05 comparing TAK to GCA with a score of 6 or higher

^***^p < 0.05 comparing GCA with a score of 6 or higher to TAK

^a^The presence of lesions of the subclavian and/or axillary arteries on both the left and right sides. The case in which one artery was the subclavian artery and the other was the axillary artery was also included

^b^The involvement of both the descending thoracic aorta and the abdominal aorta

## Discussion

In the modified version of the 2022 ACR/EULAR GCA classification criteria used in our cohort, examination using PET-CT was not mandatory for the evaluation of aortic lesions. We evaluated descending thoracic to abdominal aortic lesions mainly by the detection of wall thickening through contrast-enhanced CT or MRI. Notably, the modified 2022 ACR/EULAR GCA classification criteria showed similar sensitivity and specificity to those of the original 2022 ACR/EULAR GCA classification criteria [10]. The discriminability between GCA and TAK by C-statistic using a set of modified criteria, excluding age, was good. These suggested that the modified 2022 ACR/EULAR GCA classification criteria without age restriction well performed to classify GCA. However, the discriminative ability of the set consisting of large-vessel lesions (bilateral axillary artery and descending thoracic-abdominal aorta) and inflammatory markers, was markedly reduced, and the addition of PMR or age to the set of large-vessel lesions improved the discriminative ability.

The DCVAS cohort showed specificity of the 2022 ACR/EULAR GCA classification criteria for a control group that included various types of vasculitis, including TAK. In another validation study of the 2022 ACR/EULAR GCA classification criteria, GCA was compared with suspected GCA as a control [19–22]. It was expected that the similar clinical presentation of GCA and TAK [6, 7] would decrease specificity when only TAK was used as a control, but the modified 2022 ACR/EULAR GCA classification criteria showed high specificity even when only TAK was used as a control. In addition, the modified criteria were valuable for classifying GCA and TAK, even when age was excluded. The results also confirmed a significant increase in sensitivity for GCA without cranial artery lesions compared with the 1990 ACR classification criteria. This observation was similar to the results obtained from the DCVAS cohort [10].

In the 2022 ACR/EULAR GCA classification criteria, patients aged 50 years or older at onset who had an elevation of inflammatory markers and lesions of both bilateral axillary arteries and descending thoracic-abdominal aorta were classified as GCA [10]. In the JPVAS cohort, a set of items including bilateral axillary artery lesions, lesions in the thoracic descending aorta to the abdominal aorta, and CRP or ESR alone (Fig. 1F, model 5) exhibited poor discriminative ability for GCA and TAK. However, this discriminative power improved after the addition of PMR (Fig. 1G, model 6), suggesting the important role of PMR in discriminating between LV-GCA without cranial signs and symptoms and TAK. This is consistent with the Giant-Cell Arteritis Actemra (GiACTA) trial entry criteria [23], which classified PMR with large-vessel involvement (detected by imaging) as indicative of active GCA.

The present study suggested that the age of onset 50 years or older was crucial for the discrimination of LV-GCA without both cranial artery lesions and PMR (Fig. 1H, model 7). Therefore, the modified 2022 ACR/EULAR GCA classification criteria were considered useful for discriminating the LV-GCA without both cranial artery lesions and PMR and the TAK with an age of onset less than 50 years.

Late-onset TAK has been recognized in Japan [14], and age restriction was not included in the Japanese diagnostic criteria for TAK [16]. The incidences of bilateral axillary arteries and descending thoracic-abdominal aorta in late-onset TAK were similar to those of LV-GCA, and the specificity of the modified 2022 ACR/EULAR GCA classification criteria decreased for the late-onset TAK. Late-onset TAK meeting the modified GCA classification criteria had cranial signs or bilateral axillary artery involvement despite having unilateral left subclavian artery involvement or ascending aorta-aortic arch involvement, which is characteristic of TAK [9, 15, 24–26]. These suggest that it might be challenging to distinguish LV-GCA without PMR and late-onset TAK in a few patients with large-vessel vasculitis with age of onset older than 50 years.

Previous studies comparing PET-CT with CT or MRI indicated that aortic lesions can be diagnosed with CT [27] or MRI [28]. In the 2023 update of the EULAR recommendations, FDG-PET is recommended as the first imaging, but MRI or CT can be alternatively used for the diagnosis of extracranial arteries [29]. In the present study, the descending thoracic and abdominal aortic lesions were mainly diagnosed by CT or MRI, and PET-CT was conducted in 70% of the patients diagnosed by CT or MRI. The good performance of the modified classification criteria supports that MRI or CT can be an alternative to FDG-PET.

The present study revealed an increased proportion of bilateral subclavian and/or axillary involvement in LV-GCA compared with TAK (Supplementary Table S1) and late-onset TAK (Table 3). In previous studies, bilateral subclavian lesions were a key finding for the definition of LV-GCA [9, 30, 31], while unilateral subclavian lesions (especially left subclavian lesions) were more common than bilateral subclavian lesions in patients with TAK [9, 15, 24–26]. The prevalence of bilateral subclavian and/or axially artery lesions was higher than that of bilateral axillary artery lesions in the epidemiological study in Japan [26], and other cohorts [10, 20, 22]. Since ultrasonography for axially arteries was not routinely conducted at the time of recruitment (2007–2014) in Japan, we may have failed to detect the presence of bilateral axillary artery lesions in GCA patients with bilateral subclavian artery lesions.

The combination of temporal and axillary artery ultrasound and clinical findings is useful in the diagnosis of GCA, and the presence of wall thickening on ultrasound with typical clinical manifestations of GCA can be diagnosed without a temporal artery biopsy [32, 33]. In addition, temporal artery biopsy is less sensitive than temporal artery ultrasound with equal specificity [3, 29]. The main limitation of the present study is the lack of data on the ultrasound findings of the temporal or axillary arteries. The prevalence of temporal artery lesions detected by biopsy in the present study was similar to the DCVAS cohorts [9] but was lower than those detected by ultrasound in previous validation cohorts in European countries [19–21]. In the axillary arteries, performing ultrasonography in all cases might result in an increased detection rate of lesions [34, 35]. In the present study, 80% of the patients with bilateral axillary artery lesions in the JPVAS cohort were diagnosed by PET-CT, and the prevalence of bilateral axially artery lesions in the present study was similar to the DCVAS cohorts [9] but was lower than those detected by ultrasound in the previous validation cohorts in European countries [19–21]. The prevalence of descending thoracic to abdominal aortic lesions is similar to the DCVAS and previous validation cohorts. The sensitivity of the classification criteria was lower in this study than in previous validation cohorts [19–21], both for GCA overall and for GCA patients without temporal artery lesions, and sensitivity may be improved by performing temporal artery and axillary artery ultrasound in all patients.

The possible other limitations of this study are discussed below. Firstly, the selection of imaging modality was at the discretion of the attending physician in the retrospective JPVAS cohort, which may have affected the sensitivity and specificity of the tested criteria. Secondly, the cohort enrolled incident cases at participating institutions affiliated with JPVAS. Thus, although the results are generalizable for Japanese patients, ethnic differences may affect the sensitivity and specificity of the classification criteria. Thirdly, in our case report forms, we did not collect information on imaging findings for the celiac artery.

In conclusion, the modified 2022 ACR/EULAR GCA classification criteria exhibited good discriminative ability for GCA and TAK including late-onset TAK without PET-CT in routine clinical practice. However, age restriction or PMR was required to distinguish GCA with lesions of bilateral axillary arteries and descending thoracic-abdominal aorta from TAK. A prospective cohort study of patients with LV-GCA and late-onset TAK is warranted in the future, with protocols for evaluation by both temporal artery and axillary artery ultrasound.

## Supplementary Information

Supplementary Material 1.

## Data Availability

All of the data supporting the conclusions of this article are included within the article.

## References

[1] Hellmich B, Agueda A, Monti S, Buttgereit F, de Boysson H, Brouwer E, et al. 2018 Update of the EULAR recommendations for the management of large vessel vasculitis. Ann Rheum Dis. 2020;79(1):19–30. 31270110 10.1136/annrheumdis-2019-215672

[2] Maz M, Chung SA, Abril A, Langford CA, Gorelik M, Guyatt G, et al. 2021 American College of Rheumatology/Vasculitis Foundation Guideline for the Management of Giant Cell Arteritis and Takayasu Arteritis. Arthritis Rheumatol. 2021;73(8):1349–65. 34235884 10.1002/art.41774 PMC12344528

[3] Monti S, Agueda AF, Luqmani RA, Buttgereit F, Cid M, Dejaco C, et al. Systematic literature review informing the 2018 update of the EULAR recommendation for the management of large vessel vasculitis: focus on giant cell arteritis. RMD Open. 2019;5(2):e001003. 31673411 10.1136/rmdopen-2019-001003 PMC6803016

[4] Hunder GG, Bloch DA, Michel BA, Stevens MB, Arend WP, Calabrese LH, et al. The American College of Rheumatology 1990 criteria for the classification of giant cell arteritis. Arthritis Rheum. 1990;33(8):1122–8. 2202311 10.1002/art.1780330810

[5] Gribbons KB, Ponte C, Craven A, Robson JC, Suppiah R, Luqmani R, et al. Diagnostic assessment strategies and disease subsets in giant cell arteritis: data from an international observational cohort. Arthritis Rheumatol. 2020;72(4):667–76. 31729185 10.1002/art.41165 PMC7113106

[6] Grayson PC, Maksimowicz-McKinnon K, Clark TM, Tomasson G, Cuthbertson D, Carette S, et al. Distribution of arterial lesions in Takayasu’s arteritis and giant cell arteritis. Ann Rheum Dis. 2012;71(8):1329–34. 22328740 10.1136/annrheumdis-2011-200795 PMC3729734

[7] Maksimowicz-McKinnon K, Clark TM, Hoffman GS. Takayasu arteritis and giant cell arteritis: a spectrum within the same disease? Medicine (Baltimore). 2009;88(4):221–6. 19593227 10.1097/MD.0b013e3181af70c1

[8] Michailidou D, Rosenblum JS, Rimland CA, Marko J, Ahlman MA, Grayson PC. Clinical symptoms and associated vascular imaging findings in Takayasu’s arteritis compared to giant cell arteritis. Ann Rheum Dis. 2020;79(2):262–7. 31649025 10.1136/annrheumdis-2019-216145 PMC12207754

[9] Gribbons KB, Ponte C, Carette S, Craven A, Cuthbertson D, Hoffman GS, et al. Patterns of arterial disease in Takayasu Arteritis and Giant Cell Arteritis. Arthritis Care Res (Hoboken). 2020;72(11):1615–24. 31444857 10.1002/acr.24055 PMC7035996

[10] Ponte C, Grayson PC, Robson JC, Suppiah R, Gribbons KB, Judge A, et al. 2022 American College of Rheumatology/EULAR Classification Criteria for Giant Cell Arteritis. Arthritis Rheumatol. 2022;74(12):1881–9. 36350123 10.1002/art.42325

[11] Sugihara T, Hasegawa H, Uchida HA, Yoshifuji H, Watanabe Y, Amiya E, et al. Associated factors of poor treatment outcomes in patients with giant cell arteritis: clinical implication of large vessel lesions. Arthritis Res Ther. 2020;22(1):72. 32264967 10.1186/s13075-020-02171-6 PMC7137303

[12] Sugihara T, Uchida HA, Yoshifuji H, Maejima Y, Naniwa T, Katsumata Y, et al. Association between the patterns of large-vessel lesions and treatment outcomes in patients with large-vessel giant cell arteritis. Mod Rheumatol. 2023;33(6):1145–53. 36218378 10.1093/mr/roac122

[13] Uchida HA, Nakaoka Y, Sugihara T, Yoshifuji H, Maejima Y, Watanabe Y, et al. Clinical characteristics and treatment outcomes of patients with newly diagnosed Takayasu arteritis in japan during the first 2 years of treatment- a nationwide retrospective cohort study. Circ J. 2024. online ahead of print. 10.1253/circj.CJ-24-0178 39261026

[14] Watanabe Y, Miyata T, Tanemoto K. Current clinical features of new patients with Takayasu Arteritis observed from cross-country research in Japan: age and sex specificity. Circulation. 2015;132(18):1701–9. 26354799 10.1161/CIRCULATIONAHA.114.012547

[15] Yoshifuji H, Nakaoka Y, Uchida HA, Sugihara T, Watanabe Y, Funakoshi S, et al. Organ damage and quality of life in Takayasu Arteritis - evidence from a national registry analysis. Circ J. 2024;88(3):285–94. 38123296 10.1253/circj.CJ-23-0656

[16] Isobe M, Amano K, Arimura Y, Ishizu A, Ito S, Kaname S, et al. JCS 2017 guideline on management of vasculitis syndrome- digest version. Circ J. 2020;84(2):299–359. 31956163 10.1253/circj.CJ-19-0773

[17] Grayson PC, Ponte C, Suppiah R, Robson JC, Gribbons KB, Judge A, et al. 2022 American College of Rheumatology/EULAR classification criteria for Takayasu arteritis. Ann Rheum Dis. 2022;81(12):1654–60. 36351705 10.1136/ard-2022-223482

[18] Sugihara T, Ishizaki T, Hosoya T, Iga S, Yokoyama W, Hirano F, et al. Structural and functional outcomes of a therapeutic strategy targeting low disease activity in patients with elderly-onset rheumatoid arthritis: a prospective cohort study (CRANE). Rheumatology (Oxford). 2015;54(5):798–807. 25296748 10.1093/rheumatology/keu395

[19] Narvaez J, Estrada P, Vidal-Montal P, Nolla JM. Performance of the new 2022 ACR/EULAR classification criteria for giant cell arteritis in clinical practice in relation to its clinical phenotypes. Autoimmun Rev. 2023;22(10):103413. 37598876 10.1016/j.autrev.2023.103413

[20] Molina-Collada J, Castrejon I, Monjo I, Fernandez-Fernandez E, Torres Ortiz G, Alvaro-Gracia JM, et al. Performance of the 2022 ACR/EULAR giant cell arteritis classification criteria for diagnosis in patients with suspected giant cell arteritis in routine clinical care. RMD Open. 2023;9(2):e002970. 37094980 10.1136/rmdopen-2022-002970 PMC10151996

[21] van Nieuwland M, van Bon L, Vermeer M, Brouwer E, Alves C. External validation of the 2022 ACR/EULAR classification criteria in patients with suspected giant cell arteritis in a Dutch fast-track clinic. RMD Open. 2023;9(3):e003080. 37507207 10.1136/rmdopen-2023-003080 PMC10387624

[22] Hemmig AK, Aschwanden M, Imfeld S, Berger CT, Daikeler T. A diagnostic performance study of the 2022 American College of Rheumatology/EULAR classification criteria for giant cell arteritis in a cohort of patients presenting with suspected giant cell arteritis. Arthritis Rheumatol. 2023;75(6):1075–7. 36622332 10.1002/art.42440

[23] Stone JH, Tuckwell K, Dimonaco S, Klearman M, Aringer M, Blockmans D, et al. Trial of tocilizumab in giant-cell arteritis. N Engl J Med. 2017;377(4):317–28. 28745999 10.1056/NEJMoa1613849

[24] Mutoh T, Shirai T, Fujii H, Ishii T, Harigae H. Insufficient use of corticosteroids without immunosuppressants results in higher relapse rates in Takayasu Arteritis. J Rheumatol. 2020;47(2):255–63. 31092708 10.3899/jrheum.181219

[25] Gudbrandsson B, Molberg O, Garen T, Palm O. Prevalence, incidence, and disease characteristics of Takayasu arteritis by ethnic background: data from a large, population-based cohort resident in Southern Norway. Arthritis Care Res (Hoboken). 2017;69(2):278–85. 27159262 10.1002/acr.22931

[26] Konda N, Sakai R, Saeki K, Matsubara Y, Nakamura Y, Miyamae T, et al. Nationwide clinical and epidemiological study of large-vessel vasculitis in Japan in 2017. Mod Rheumatol. 2023;34(1):167–74. 36737863 10.1093/mr/road019

[27] de Boysson H, Dumont A, Liozon E, Lambert M, Boutemy J, Maigne G, et al. Giant-cell arteritis: concordance study between aortic CT angiography and FDG-PET/CT in detection of large-vessel involvement. Eur J Nucl Med Mol Imaging. 2017;44(13):2274–9. 28736805 10.1007/s00259-017-3774-5

[28] Quinn KA, Ahlman MA, Malayeri AA, Marko J, Civelek AC, Rosenblum JS, et al. Comparison of magnetic resonance angiography and (18)F-fluorodeoxyglucose positron emission tomography in large-vessel vasculitis. Ann Rheum Dis. 2018;77(8):1165–71. 29666047 10.1136/annrheumdis-2018-213102 PMC6045453

[29] Dejaco C, Ramiro S, Bond M, Bosch P, Ponte C, Mackie SL, et al. EULAR recommendations for the use of imaging in large vessel vasculitis in clinical practice: 2023 update. Ann Rheum Dis. 2024;83(6):741–51. 37550004 10.1136/ard-2023-224543

[30] Brack A, Martinez-Taboada V, Stanson A, Goronzy JJ, Weyand CM. Disease pattern in cranial and large-vessel giant cell arteritis. Arthritis Rheum. 1999;42(2):311–7. 10025926 10.1002/1529-0131(199902)42:2<311::AID-ANR14>3.0.CO;2-F

[31] Muratore F, Kermani TA, Crowson CS, Green AB, Salvarani C, Matteson EL, et al. Large-vessel giant cell arteritis: a cohort study. Rheumatology (Oxford). 2015;54(3):463–70. 25193809 10.1093/rheumatology/keu329 PMC4425829

[32] Sebastian A, van der Geest KSM, Tomelleri A, Macchioni P, Klinowski G, Salvarani C, et al. Development of a diagnostic prediction model for giant cell arteritis by sequential application of Southend Giant Cell Arteritis Probability Score and ultrasonography: a prospective multicentre study. Lancet Rheumatol. 2024;6(5):e291–9. 38554720 10.1016/S2665-9913(24)00027-4

[33] Molina Collada J, Martinez-Barrio J, Serrano-Benavente B, Castrejon I, Caballero Motta LR, TrivesFolguera L, et al. Diagnostic value of ultrasound halo count and Halo Score in giant cell arteritis: a retrospective study from routine care. Ann Rheum Dis. 2022;81(9):e175. 32759266 10.1136/annrheumdis-2020-218631

[34] Hop H, Mulder DJ, Sandovici M, Glaudemans A, van Roon AM, Slart R, et al. Diagnostic value of axillary artery ultrasound in patients with suspected giant cell arteritis. Rheumatology (Oxford). 2020;59(12):3676–84. 32240306 10.1093/rheumatology/keaa102 PMC7733725

[35] Bull Haaversen AC, Brekke LK, Kermani TA, Molberg O, Diamantopoulos AP. Extended ultrasound examination identifies more large vessel involvement in patients with giant cell arteritis. Rheumatology (Oxford). 2023;62(5):1887–94. 35997556 10.1093/rheumatology/keac478

