# Supplementary material for: Performance of the modified 2022 ACR/EULAR giant cell arteritis classification criteria without age restriction for discriminating from Takayasu arteritis
Source: Arthritis Res Ther. 2025 Jan 31;27:19. doi: 10.1186/s13075-025-03486-y (PMC11783758; doi:10.1186/s13075-025-03486-y)
Supplement: Supplementary file 1 — Supplementary Material 1. [file 13075_2025_3486_MOESM1_ESM.docx]

Supplementary Table S1 Comparison of clinical parameters of large-vessel involvement included in the modified 2022 ACR/EULAR GCA classification criteria in patients with LV-GCA and TAK

|  | LV-GCA (n=73) | TAK (n=129) | *P*-value | OR (95%CI) |
| --- | --- | --- | --- | --- |
| Left axially artery, % | 20.5 | 12.4 | 0.123 | 1.83  (0.84–3.95) |
| Right axially artery, % | 16.4 | 7.0 | 0.034 | 2.62  (1.05–6.57) |
| Bilateral axillary artery, % | 13.7 | 6.2 | 0.072 | 2.40  (0.90–6.39) |
| Left subclavian artery, % | 56.2 | 64.3 | 0.252 | 0.71  (0.40–1.28) |
| Right subclavian artery, % | 45.2 | 36.4 | 0.221 | 1.44  (0.80–2.58) |
| Bilateral subclavian and/or axillary artery^*^, % | 41.1 | 30.2 | 0.118 | 1.61  (0.89–2.93) |
| Left or right subclavian and/or axillary artery, % | 60.3 | 70.5 | 0.136 | 0.63  (0.35–1.16) |
| Descending thoracic aorta-abdominal aorta^**^, % | 45.2 | 32.6 | 0.074 | 1.71  (0.95–3.08) |
| Ascending aorta | 31.5 | 48.8 | 0.017 | 0.48  (0.26–0.88) |
| Aortic arch | 47.9 | 57.4 | <0.001 | 0.69  (0.39–1.23) |
| Aortic involvement of ≥ two lesions | 52.1 | 58.9 | 0.345 | 0.76  (0.43–1.35) |

Imaging examinations of large-vessel lesions were performed in 135 of the 139 patients at diagnosis of GCA, and 73 had large-vessel lesions as diagnosis of LV-GCA. Categorical variables were examined by using the chi-squared test. Odds ratios of being diagnosed with LV-GCA compared to TAK were calculated.

^*^The presence of lesions of the subclavian and/or axillary arteries on both the left and right sides. The case in which one artery was the subclavian artery and the other was the axillary artery was also included.

^**^The involvement of both the descending thoracic aorta and the abdominal aorta

LV-GCA, large-vessel GCA; OR, odds ratios; TAK, Takayasu arteritis.
